# Multilevel Analysis of Community Acquired Pneumonia Among 2‐59 Months Old Children in Awi Zone, Northwest Ethiopia: A Community‐Based Cross‐Sectional Study

**DOI:** 10.1002/hsr2.71375

**Published:** 2025-10-13

**Authors:** Nigussie Adam Birhan, Alene Yirsaw Workineh, Gedif Mulat Alemayehu, Zelalem Meraf Wolde, Chalachew Alemie Messfin

**Affiliations:** ^1^ Department of Statistics, College of Natural and Computational Science Injibara University Injibara Amhara Regional State Ethiopia; ^2^ Injibara General Hospital Injibara Amhara Regional State Ethiopia; ^3^ One Acre Fund Bahir Dar Amhara Regional State Ethiopia

**Keywords:** associated factor, community acquired pneumonia, prevalence, under‐five children, upper respiratory tract infection

## Abstract

**Background and Aims:**

Worldwide, pneumonia was responsible for about 740,180 deaths in children under 5 year, accounting for 14% of all deaths in 2019. The purpose of this study was to identify associated risk factors of community acquired pneumonia among 2–59 months old children in Awi Zone, Northwest Ethiopia.

**Methods:**

A community‐based cross‐sectional study was conducted from March to July 2023, using a multistage sampling method to select 1368 participants. Data were collected using a structured questionnaire. Multilevel analysis was used to identify factors associated with community‐acquired pneumonia. Variables with *p*‐value < 0.05 were considered as statistically significant.

**Results:**

The prevalence of community acquired pneumonia among 2‐59 months old was 11.33%. Primary education (AOR = 0.34; 95% CI: 0.12, 0.95), stunting (AOR = 6.12; 95% CI: 2.41, 15.51), having diarrhea (AOR = 5.04; 95% CI: 2.41, 10.55), history of acute lower respiratory tract infection (AOR = 20.84; 95% CI: 3.95, 109.83), use of charcoal as fuel source (AOR = 130.56, 95% CI: 5.94, 2869.18), carrying of a child on mother during cooking (AOR = 2.27, 95% CI: 1.10, 4.69), and presence of separate kitchen (AOR = 0.38; 95% CI: 0.19, 0.74) were associated with community acquired pneumonia.

**Conclusion:**

Mother education, age of child, stunting, presence of separate kitchen, previous respiratory tract infection, use of charcoal for fuel source, carrying of a child on mother during cooking, and history of diarrhea showed a significant association with community acquired pneumonia. Therefore, we recommend adequate health education on nutrition, diarrhea prevention and treatment, and reducing indoor air pollution to reduce the risk of community‐acquired pneumonia.

**Abbreviations**
ALRTI: acute lower respiratory tract infection
AOR: adjusted odds ratio
AURTI: acute upper respiratory tract infection
CAP: community acquired pneumonia
CI: confidence interval
COR: crude odds ratio
FMoH: Federal Ministry of Health
WHO: World Health Organization

## 1 Introduction

Community‐acquired pneumonia (CAP) is a lower respiratory tract infection that causes inflammation of the pulmonary parenchyma [1]. It can be caused by bacteria, viruses, fungi, or parasitic [2]. The presence of cough with rapid or difficult breathing, based on age‐specific criteria, is used to classify suspected pneumonia in children under 5 years old [3, 4].

According to World Health Organization, rapid breathing is characterized by a respiratory rate exceeding 60 breaths/min in newborns ( < 2 months), > 50 breaths/minute in infants (2–12 months), and > 40 breaths/minute in children (12–59 months) [5].

CAP remains a leading cause of morbidity and mortality in both developed and developing countries [6]. In 2019, it was responsible for about 740,180 deaths in children under 5 year, accounting for 14% of all deaths globally [7].

In sub‐Saharan Africa, approximately 4 million pneumonia cases occur each year, leading to about 200,000 deaths [8]. In low‐income nations, pneumonia is the primary cause of childhood mortality and accounts for about 21% of all pediatric deaths [9]. In sub‐Saharan Africa, pneumonia is estimated to account for 17%–26% of child deaths [10]. Pneumonia affects over 3,370,000 children under five in Ethiopia annually, yet it is readily avoidable and treatable with simple and minimum costs [11].

In Ethiopia, pneumonia is responsible for 16.4% of deaths among children under five, which is higher than the combined rates of diarrhea, malaria, AIDS, and measles [12].

Numerous investigations have been carried out in Ethiopia and other countries to identify the factors that contribute to CAP [9, 13, 14, 15, 16, 17]. However, these studies were done using standard regression model by omitting community level effects. Most standard regression models operate under the assumption of independence among clustered observations. However, this assumption is often violated in datasets where observations are hierarchical in nature. Estimating the actual impact of factors influencing CAP is therefore crucial, as is evaluating variations both within and between districts. Hence, researchers have attempted to determine whether there was a significant variation across the districts of Awi zone that helps to implement more effective future health care policies like resource allocation and access to health facilities which focus on specific subjects at several levels of the hierarchy nature, and there might be a correlation between the community levels using multilevel analysis.

The findings of this study have the potential benefit for stakeholders, including policymakers, organizations working on pneumonia, and future researchers. Moreover, it provides valuable evidence to inform the planning and implementation of interventions aimed at preventing CAP and reducing its impact. Therefore, this study aimed to identify risk factors for CAP and to assess the extent of variation between districts among children aged 2–59 months in Awi Zone, Northwest Ethiopia, using multilevel analysis.

## 2 Methods and Materials

### 2.1 Study Area and Period

A community‐based cross sectional study was carried out from March to June, 2023 in Awi zone, Northwest Ethiopia. Its administrative center, Injibara, is located in northwest part of Ethiopia, 114 km away from Bahir Dar, the capital of Amhara regional state, and 449 km northwest of the capital city of Ethiopia, Addis Ababa. According to the 2007 census conducted by the Ethiopian Central Statistical Agency, it has a total population of 982,942 of whom 491,865 are men and 491,077 women [18]. The zone has total of fifteen districts, six urban and nine rural.

### 2.2 Study and Source Population

All children aged 2–59 months residing in the Awi Zone were considered as the source population, while those within the selected households were taken as the study population.

### 2.3 Sample Size and Sampling Technique

**Figure 1:**
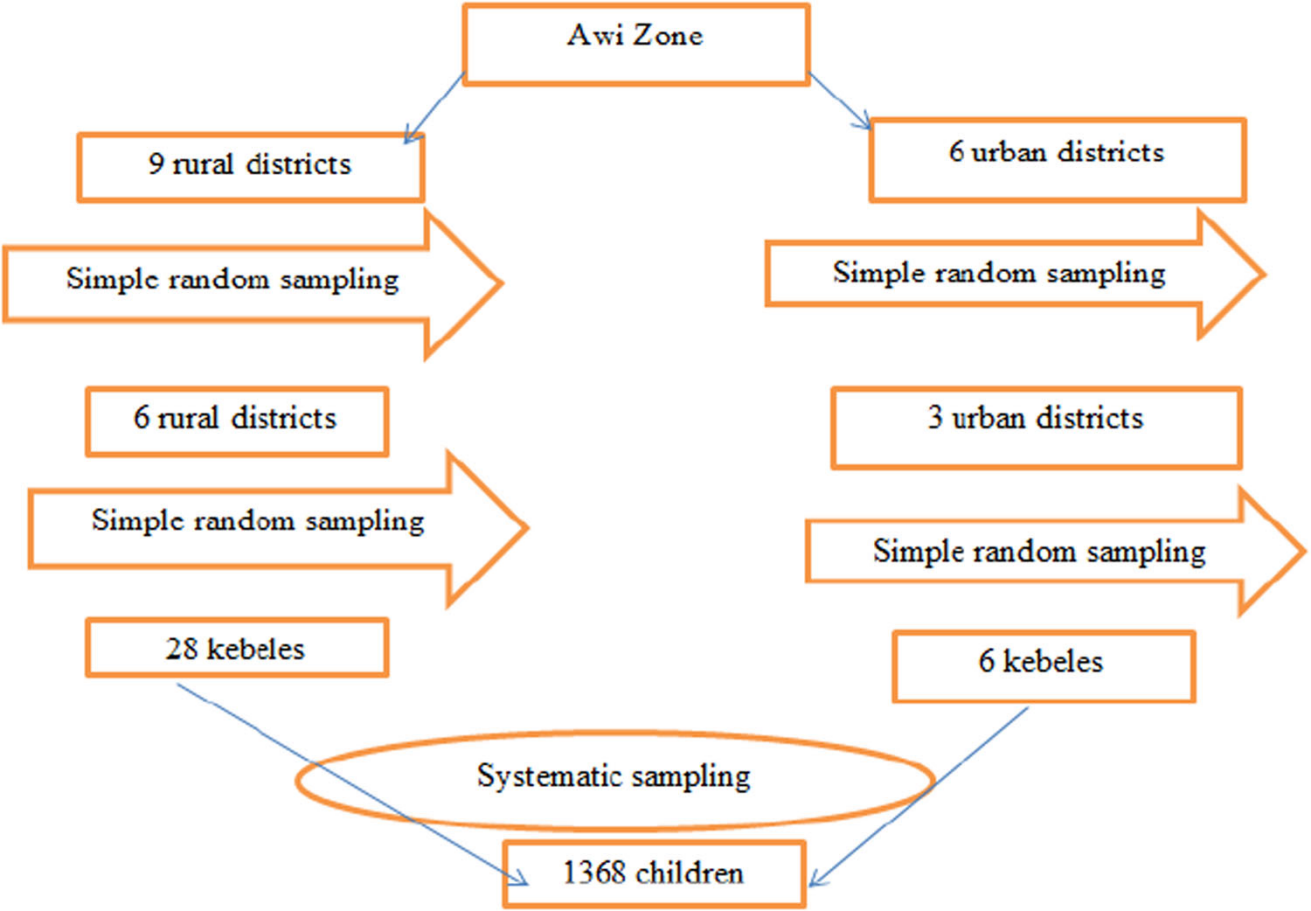
Schematic diagram indicating sampling procedure.

The sample size was calculated using a single population proportion formula, based on prevalence of pneumonia (17.7%) reported in a previous study from Munesa District, Arsi Zone, Oromia region, Ethiopia [19], a confidence level of 95% and a margin error of 3%, and consider a design effect of 2 as we plan to use multi‐stage cluster sampling technique. Hence, the final sample size was 1368 including a 10% nonresponse rate. Multistage sampling technique was employed to select study participants among the 15 districts. Nine districts (three urban and six rural districts) were selected using simple random sampling technique. Similarly, 34 kebeles (smallest administrative unit) from each district were chosen randomly in second stage. Finally, systematic sampling technique was used to select 1368 study participants using a probability proportional to the population size (Figure 1).

### 2.4 Data Collection Technique and Quality Control

Data were collected using a pretested, interviewer‐administered structured questionnaire. The questionnaire was initially prepared in English and translated in to Amharic, and then back to English to ensure consistency. Primary data were collected by eight data collectors and two experienced supervisors with degree holders in public health officers and nurses using the Amharic version of the questionnaire. Three day training was given for data collectors and supervisors on the study's goals, how to choose study participants, how to conduct interviews, how to obtain consent, how to handle data, how to maintain quality of data, and ethical issue. The questionnaire was pre‐tested on 5% of study subjects before the actual survey in kebeles was not included in the main survey and proper categorization and coding of the data collection instrument before conducting the study.

### 2.5 Study Variable

#### 2.5.1 Response Variable

The response variable was the occurrence of CAP which is defined as acute infection of lower respiratory tract infection for < 2 weeks duration, acquired in the community which leads to cough, fast breathing, and fever. This was a binary response variable coded as “Yes = 1” and “No = 0.”

#### 2.5.2 Risk Factor

**Figure 2:**
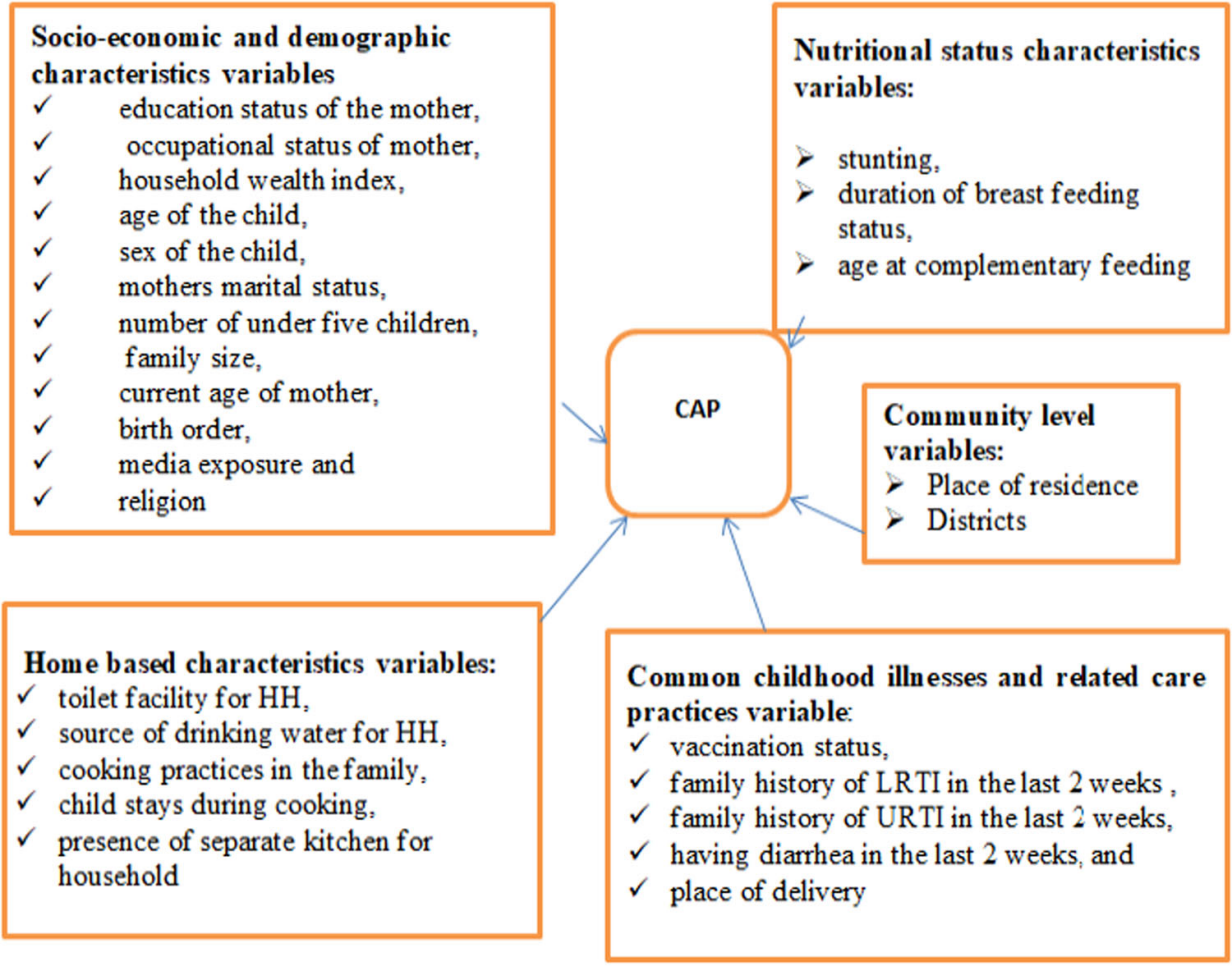
Covariates included in the model.

Based on the global and local literature reviews, various socioeconomic and demographic variables, nutritional status characteristics variable, common childhood illnesses and related care practices variable and home based characteristics were included as predictor variables (Figure 2).

##### 2.5.2.1 Operational Definition

Household economic status was assessed using a wealth index based on household assets, housing characteristics, water source, and sanitation facilities. The index was constructed using principal component analysis (PCA), and households were ranked and divided into quintiles. Following standard practice, the lowest two quintiles were classified as “poor,” the middle quintile as “middle,” and the highest two quintiles as “rich” [20].

### 2.6 Data Management and Method of Analysis

The data consistency and completeness were checked, and then variables were coded by the principal investigators, and entered in to Epi‐Data version 4.2, and exported to STATA version18 for further analysis. The descriptive statistics were computed and analyses were presented using frequency tables and percentages. The study was reported in accordance with the strengthening the reporting of observational studies in Epidemiology (STROBE) guidelines for observational studies [21].

A multilevel logistic regression model with random intercepts was used to account for the hierarchical structure of the data (children nested within districts). The model estimated both fixed effects of individual/household‐level predictors and random effects to capture unexplained variability across districts.

To solve the analytical issues that come up when data is arranged hierarchically, with children nested in districts, multilevel models were employed. Variables associated with CAP were first evaluated using a bi‐variable multilevel logistic regression model, with variables showing a p‐value less than 0.25 considered for multivariable multilevel logistic regression model [22]. Model comparison and fitness were assessed based on the likelihood ratio test (LRT) and deviance (‐2LLR) values and the model with the minimum deviance value is considered as a better fit [23]. Finally adjusted odds ratio (AOR) with a 95% confidence interval was reported for the best‐fitted model.

Four models were fitted; null model containing no explanatory variables which was used to check variation in community and provide evidence to assess random effects at the community level. Model I was the multivariable model adjustment for individual‐level variables and model II was adjusted for community‐level factors. In model III adjusted for both individual and community‐level variables were fitted with the community acquired pneumonia.

The fixed effects were used to estimate the association between the likelihood of community acquired pneumonia and explanatory variables. The result was expressed as AOR with 95% confidence interval. Regarding to random‐effects, intra cluster correlation coefficient (ICC) [24], percentage change in variance (PCV), and median odds ratio were used. The intra class correlation coefficient (ICC) was calculated to quantify the proportion of variance attributable to district‐level differences. When logistic regression model is used the residual at level one (child level) are assumed to follow the standard logistic distribution with mean 0 and variance $\frac{\pi^{2}}{3}=3.29.$ It is expressed as: $\mathrm{ICC}=\frac{\sigma_{\mu 0}^{2}}{\sigma_{\mu 0}^{2}+\frac{\pi^{2}}{3}}$, $\sigma_{\mu 0}^{2}$ is the variance of the higher level (Community).

### 2.7 Ethics Approval and Consent to Participate

The study was conducted after obtaining ethical clearance from Injibara University, College of Natural and Computational Sciences, Research and Community Service Vice Dean, under reference number CNCS/PG/R/CS/V/Dean/255/23. A formal letter of permission to carry out the study was also secured from the administrative offices involved. Verbal and written informed consent was obtained from parents or legal guardians of all eligible children after the purpose of the study was clearly explained. Participation was entirely voluntary, and an individual who does not take part had their decision fully respected. All collected data were kept confidential and anonymous, with no access granted to third parties. All the procedures of this study were conducted according with relevant guidelines and regulations.

## 3 Results

### 3.1 The Prevalence of Community Acquired Pneumonia in Awi Zone

One hundred fifty five (11.33%) (95% CI: 9.48%–12.81%) of the children had experienced community acquired pneumonia in the 2 weeks preceding the study. About 17.18% of 2–59 months old children were from Jawi district and majority 895 (64.42%) of 2–59 months old children were living in rural areas (Table 1).

**Table 1:** Community level characteristics of the study population in Awi zone.

Variables	Categories	Frequency	Percent
Residence	Urban	473	34.58
	Rural	895	65.42
District	Ankesha	140	10.23
	Ayu Guagusa	210	15.35
	Chagi city administration	157	11.48
	Injibara city administration	86	6.29
	Dangila city administration	64	4.68
	Dangila	182	13.30
	Figita lekoma	197	14.40
	Gagusa	97	7.09
	Jawi	235	17.18

### 3.2 Results of the Socio‐Demographic Characteristics of Respondents

In this study, nearly 43% of the respondents were classified as poor wealth index based on the defined socioeconomic criteria. A majority of respondents, 1300 (95.03%) were married. 299 (21.86%) held secondary and above levels of education and 725 (53.00%) had no formal education. A majority of the respondents 766 (55.99%) had a family size of five and above and 761 (55.63%) of respondents were farmers (Table 2).

**Table 2:** Socio demographic characteristics of respondents in Awi zone.

Variables	Categories	Frequency	Percentage
Sex of child	Male	771	56.36
	Female	597	43.64
Religion	Orthodox	1287	94.08
	Other^*c^	81	5.92
Educational status of mother	No education	725	53.00
	Primary	344	25.15
	Secondary and above	299	21.86
Marital status	Married	1300	95.03
	Other^*a^	68	4.97
Main occupation of mothers	Farmer	761	55.63
	Government employed	77	5.63
	Housewife	450	32.89
	Other^*b^	80	5.85
Age of mother	24 and below	124	9.06
	25–34	736	53.80
	35 and above	508	37.13
Child birth order	First	332	24.27
	2–3	506	36.99
	4–5	324	23.68
	6 and above	206	15.06
Family size of house hold	Less than five	602	44.01
	Five and above	766	55.99
Number of under five children in the family	One	1035	75.66
	Two and above	333	24.34
Age of the child in months	2–11	327	23.90
	12–23	382	27.92
	24–35	270	19.74
	36–59	389	28.44
Wealth index of house hold	Poor	581	42.47
	Middle	273	19.96
	Rich	514	37.57
Media exposure	No	662	48.39
	Yes	706	51.61

*Note:* other^*a^ = single, divorced, and widowed; other^*b^ = private employee, NGO employee and daily laborer; other^*c^ = muslim, and protestant.

Among the study participant, 1034 (75.58%) have separate kitchen for cooking, and only 24 (1.75%) have electricity for cooking, and 911 (66.59%) child stay on mother back during cooking. Concerning the toilet availability, 1268 (92.69%) of the households have toilet facility (Table 3).

**Table 3:** Home based characteristics of respondents in Awi zone.

Variables	Categories	Frequency	Percentage
Fuel mainly used for Cooking	Wood	1063	77.70
	Charcoal	281	20.54
	Electricity	24	1.75
Child stays during cooking	Out of cooking room	457	33.41
	On mother back	911	66.59
Presence of separate kitchen for household	No	334	24.42
	yes	1034	75.58
Presence of window	No	139	10.16
	Yes	1229	89.84
Main source of drinking water	Unprotected	225	16.45
	Protected	1143	83.55
Toilet availability of family	No	100	7.31
	Yes	1268	92.69

Among the total participants, about 939 (68.64%) of children breast feed greater than 12 months and 354 (25.88%) were stunted, whereas 1014 (74.12%) of them are normal (Table 4).

**Table 4:** Nutritional status characteristics of respondents in Awi zone.

Variables	Categories	Frequency	Percentage
Duration of breast feeding status	12 and less than months	429	31.36
	Greater than 12 month	939	68.64
Nutritional status of the child	Normal	1014	74.12
	Stunting	354	25.88
Beginning of complementary feeding at 6 months	Not starting	116	8.48
	No	541	39.55
	Yes	711	51.97

According to this study, about 1086 (79.39%) children were fully vaccinated and 234 (17.11%) had no history of diarrhea (Table 5).

**Table 5:** Common childhood illnesses and related care practices of respondents in Awi zone.

Variables	Categories	Frequency	Percentage
History of lower respiratory tract infection in the last 2 weeks	No	1222	89.33
	Yes	146	10.67
History of upper respiratory tract infection in the last 2 weeks	No	1212	88.60
	Yes	156	10.40
Child vaccination status	Partially	282	20.61
	Fully	1086	79.39
History of having diarrhea in the last 2 weeks	No	1134	82.89
	Yes	234	17.11
Place of delivery	Home	90	6.58
	Health institution	1278	93.42

### 3.3 Test of Heterogeneity

It is necessary to check for the heterogeneity of CAP among children aged 2–59 months in the districts of the Awi zone before beginning the multilevel analysis. A chi‐square Pearson test = 92.43 with *p*‐value < 0.001 to be statistically significant at levels. Thus, there is strong evidence for variation among the districts, with respect to the CAP among children aged 2–59 months in Awi Zone.

### 3.4 Risk Factors for Community‐Acquired Pneumonia Among Children Aged 2–59 Months

The model comparison results indicated that Model III provided the best fit for the data among the evaluated models, as it had the lowest Deviance statistic (Table 7). In the final model; child's age, mother's education status, stunting status, having diarrhea in the past 2 weeks, family history of lower respiratory tract infection in the last 2 weeks, family history of upper respiratory tract infection in the past 2 weeks, fuel mainly used for Cooking, child's location during cooking, and presence of separate kitchen in the household were the significant determinant of CAP among children 2–59 months. Children in the age groups of 12–23, 24–35, and 36–59 months were 0.03 times (AOR = 0.03; 95% CI: 0.01, 0.10), 0.39 times (AOR = 0.39; 95% CI: 0.16, 0.93), and 0.16 times (AOR = 0.16; 95% CI: 0.05, 0.45) less likely to develop community acquired pneumonia as compared to the age group of 2–11 months, respectively

This study showed that children from households with a history of acute lower respiratory tract infection in the last 2 weeks before survey date were 20.84 times [AOR = 20.84; 95% CI: 3.95, 109.83] more likely to develop community acquired pneumonia as compared to its counterparts. Similarly, children from households with a history of acute upper respiratory tract infection in the past 2 weeks before survey date were 11.11 times [AOR = 11.11; 95% CI: 2.18, 56.75] more likely to develop CAP as compared to their counterparts.

Children who experienced diarrhea during the past 2 weeks before the survey date were five times (AOR = 5.04; 95% CI: 2.41, 10.55) more likely to develop CAP compared to their counterparts. The estimated odds of having CAP for children whose mother attained primary education were 0.34 times less likely as compared to women who did not attained education (AOR = 0.34; 95% CI: 0.12, 0.95). Likewise, the odds of developing CAP in children were 6 times (AOR = 6.12; 95% CI: 2.41, 15.51) more likely among stunting children than normal children. Children from households that use charcoal as the primary cooking fuel were 131 times more likely to develop CAP compared to those from households using electricity (AOR = 130.56; 95% CI: 5.94–2869.18). On the other hand, child who was carried on the mother back during cooking was two times more likely to develop CAP as compared to children outside of the cooking room (AOR = 2.27, 95% CI: 1.10, 4.69).

This study found that children from households with a separate kitchen for cooking had a 62% reduced risk of developing CAP compared to those from households without a separate kitchen (AOR = 0.38; 95% CI: 0.19, 0.74) [Table 6].

**Table 6:** Bivariable and multivariable analysis of factors associated with community acquired pneumonia among 2–59 months old children in Awi zone.

Variables	Categories	COR (95% CI)	AOR (95% CI)	*p*‐value
Child's age in months	2–11	1	1	
	12–23	0.39 (0.24, 0.63)	0.30 (0.01, 0.10)	< 0.001*
	24–35	0.57 (0.36, 0.90)	0.39 0.16, 0.93)	0.04*
	36–59	0.38 (0.24, 0.60)	0.16 (0.05, 0.45)	< 0.001*
Educational level of mother/caregiver/	No education	1	1	
	primary	0.67 (0.44,1.04)	0.34 (0.12, 0.95)	0.04*
	Secondary and above	0.94 (0.62,1.42)	2.40 (0.85, 6.77)	0.10
Media exposure	No	1	1	
	Yes	0.70 (0.50,0.99)	1.30 (0.57, 2.97)	0.53
Main source of drinking water	Unprotected	1	1	
	Protected	0.64 (0.42,0.96)	0.74 (0.30, 1.85)	0.52
Wealth index of family	Poor	1	1	
	Middle	0.68 (0.43,1.06)	0.51 (0.19, 1.36)	0.18
	Rich	0.54 (0.37,0.80)	1.09 (0.38, 3.13)	0.87
Religion	Orthodox	1	1	
	Other^*c^	0.29 (0.09,0.92)	0.20 (0.03, 1.24)	0.08
Nutritional status	Normal	1	1	
	Stunting	1.52 (1.06,2.18)	6.12 (2.41, 15.51)	< 0.001*
Place of delivery	Home	1	1	
	Health facility	0.56 (0.32,0.99)	2.81 (0.71, 11.14)	0.14
Child initiated complementary feeding at 6 month	No	1	1	
	Yes	0.73 (0.52,1.03)	0.97 (0.52, 1.82)	0.93
	No starting	0.28 (0.11,0.71)	0.99 (0.17, 5.66)	0.99
Child vaccination	Partially	1	1	
	Fully	0.69 (0.47,1.01)	0.58 (0.24, 1.41)	0.23
Having diarrhea in the last 2 weeks	No	1	1	
	Yes	5.82 (4.07,8.31)	5.04 (2.41, 10.55)	< 0.001*
History of acute lower respiratory tract infection in the last 2 weeks	No	1	1	
	Yes	102.62 (62.20,169.30)	20.84 (3.95, 109.83)	< 0.001*
History of acute upper respiratory tract infection in the last 2 weeks	No	1	1	
	Yes	98.61 (60.37,161.07)	11.11 (2.18, 56.75)	0.004*
Fuel mainly used for Cooking	Electricity	1	1	
	Charcoal	1.48 (0.19, 11.64)	130.56 (5.94, 2869.15)	0.002*
	Wood	3.40 (0.46, 25.40)	500.40 (20.47, 12233.82)	< 0.001*
Child stays during cooking	Out of cooking room	1	1	
	On mother back	1.45 (1.00, 2.11)	2.27 (1.10, 4.69)	0.03*
Presence of separate kitchen for household	No	1	1	
	Yes	0.45 (0.32, 0.63)	0.38 (0.19, 0.74)	0.005*
Presence of window	No	1	1	
	Yes	0.48 (0.30, 0.76)	0.97 (0.36, 2.63)	0.96
Residence	Urban	1	1	
	Rural	1.26 (0.87, 1.83)	1.33 (0.53, 3.29)	0.54

*Note:* 1 reference group; other^*c^ = muslim, and protestant.

* Significance at *p*‐value < 0.05.

### 3.5 Random Effect Analysis

The ICC of 16.7% in the null model indicated that a total variation in community acquired pneumonia prevalence among 2–59 months old children was attributable to differences between districts in Awi zone. After adjusting for other variables, the between‐district variation remained significant, suggesting potential unmeasured district‐level factors. The MOR of 1.680 in the null model showed that there is a higher clustering of community acquired pneumonia among 2–59 months old children. Although we adjusted for several known risk factors, the PCV of 48.02% indicates that a notable proportion of the variation remains unexplained, potentially due to unmeasured district‐level determinants such as environmental conditions, healthcare accessibility, or public health infrastructure (Table 7).

**Table 7:** Variability for community level and model fitness for CAP.

Parameter		Null model	Model I	Model II	Model III
Community level variance		0.658	0.436	0.640	0.342
ICC		0.167	0.117	0.163	0.094
MOR		1.680	1.526	1.668	1.454
PCV (%)		reference	33.74	2.74	48.02
Model fitness					
Log likehood		−457.34	−186.28	−457.17	−178.80
Deviance (‐2LL)		914.68	372.56	914.34	357.6

## 4 Discussion

The findings of this study indicated that the 2 weeks prevalence of CAP among children aged 2–59 months was 11.33% (95% CI: 9.75%–13.12%). The result was lower than studies done in University of Gondar referral hospital, Ethiopia (18.5%) [25], in Munesa district, Arsi Zone, Oromia region, Ethiopia (17.7%) [19]. However, it was higher than the finding done in Angolela Tera district, North Showa, Ethiopia (5.8%) [17]. The difference might be attributed to variations in socio demographic, behavioral, and environmental factors among the study households compared to other studies such as, difference in people's way of life, community educational levels, maternal and child healthcare practice, immunization coverage, and nutritional status.

In this study, multilevel analysis revealed district‐level clustering of community acquired pneumonia cases, suggesting that environmental or service‐related factors at the district level may contribute to the burden of disease. Cluster analysis using a chi‐square test (*χ*² = 92.43, *p* < 0.001) showed significant variation in CAP across districts in Awi Zone. Results obtained based on the empty model and the overall variance of the constant suggests that children's community acquired pneumonia differed across Awi zone. While household factors such as cooking practices and prior infections were significant, residual variation between districts points to the need for further investigation into regional health services, environmental exposures, or socioeconomic conditions. Possible drivers of these differences include variation in access to healthcare services, availability and quality of immunization and nutrition programs, differences in environmental exposures, and socioeconomic disparities, local infrastructure cultural practices.

After accounting for predictor factors the median increase in the odds of community acquired pneumonia as compared to children at a cluster with higher risk of community acquired pneumonia to children at a cluster with lower risk of community acquired pneumonia was 45.4% increased. These imply significant disparity of the problem which requires a context based interventions to tackle this cyclic intergeneration linked health problem.

The odds of developing CAP among 2–59 months old children lived in household who had history AURTI in last 15 days before survey date were nine times more likely to develop CAP as compared to children lived in household who hadn't AURTI. This finding was in agreement with the previous studies done in Oromia zone, Ethiopia [26], Kersa District, Southwest Ethiopia [27], and Kenya [28]. This can be explained that upper respiratory tract infections weaken child feeding habit by making them anorexic. This lowers the children's nutritional status and weakens their immune systems, which increases the risk of CAP [27].

Children who had a history of diarrhea in the last 2 weeks before survey date were four times more likely to develop CAP compared to their counterparts. This finding was in agreement with the other studies conducted in Tigray Ethiopia [29], in urban areas of Oromia special zone of Amhara region [26], in Hossana town, Ethiopia [15]. The possible reason could be children who have a concomitant illness like diarrhea may have a lowered immunity, making them more susceptible to diseases like pneumonia [30].

Children from household who had a past history of ALRTIs in household member in the last 2 weeks before the data collection were 15 times higher risk of developing community acquired pneumonia. The finding is consistent with a study done in Kemise, Oromia zone, Amhara region [9]. This could be due to lower respiratory tract infections were contagious and are easily transmittable from family member contacts to children. These infections are often presumed to be viral in origin based on clinical patterns reported in similar settings, and may result from the progression of milder lower respiratory tract infections, predisposing children to pneumonia. The severity of disease can also be influenced by the virulence and load of the pathogen, which is generally higher when transmitted through close household contact [31].

Regarding to stunting, children who are stunting were five times higher chance of developing community acquired pneumonia as compared to normal children. It coincides with the previous studies done in Hossana town, Ethiopia [15], Worabe town [32] and Bangladesh [33]. The possible reason might be due to the fact that stunting indicates children long term malnutrition which weakens the immune system needed to clear secretions in the respiratory tract, declines child's natural body defense mechanism and child becomes susceptible for the infection causing agent, which intern predisposes to community acquired pneumonia [34].

According to this study having a separate kitchen from the main house for cooking were significantly associated with a lower risk of community acquired pneumonia among 2‐59 months old children. Children from households with a separate kitchen were 62% less likely to develop CAP compared to those without a separate kitchen (AOR = 0.38). This finding is consistent with a study conducted in Wondogenet district in Sidama zone, Ethiopia [35]. This could be the reason that the high risk of household air pollution, which contributes to pulmonary inflammation and tissue damage, creating a favorable environment for pathogens and increase susceptibility of children to community acquire pneumonia [4].

This study showed that educational level of mother had significant effect on community acquired pneumonia. This finding had conformity with a study done in Slums of Dibrugarh town [36]. Educated mothers recognize the signs and symptoms of pneumonia early and so accesses health care earlier and so their children have a better outcome than others.

Children at older age range groups were less likely to develop community acquired pneumonia when compared to age range 2‐11 months. The result is consistent with other previous studies [14, 15]. This can be explained by the small airways and immature defense system of this age group that makes them more susceptible to develop community acquired pneumonia [37].

Furthermore, a child whose family used charcoal as main fuel source was more likely to developing community acquired pneumonia than those who did use electricity. This is in line with study conducted in Este town, Northwest Ethiopia [38], Rabat, Morocco [39]. The possible explanation for this might be families who burning charcoal produce indoor air pollutants such as carbon monoxide and fine particulate matter, which can irritate the respiratory tract and impair lung function, thereby increasing the risk of respiratory infections like pneumonia [40].

Children carried on the back of mothers or caregivers during cooking were associated with an increased risk of CAP by two times more likely to develop CAP. This result is consistent with case control study conducted in Worabe town found that a child who was carried on the back of mother during cooking was two times more likely to develop pneumonia than their counterparts [32]. Similar findings were obtained in different studies done in Este town, Ethiopia [38], and Greater Banjul and Upper River Regions, the Gambia [41]. This is because children who are in the kitchen during cooking especially on the back of the mother or caregiver expose to indoor air pollution as a result of different cooking fuels that lead to respiratory tract infection. Moreover, holding a child on back while cooking foods can increase the probability of inhaling smokes and food vapors which in turn will increase the risk of community acquired pneumonia by altering the structure and function of the respiratory tract [42, 43].

## 5 Strength and Limitation of the Study

The strength of this study was the analysis use primary data and pretest was done before the actual data collection. The limitation of this study was a cross‐sectional survey study, which may not set a temporal relationship between cause and effect. There might be a possibility of recall bias, due to limit social desirability, and interviewer effect because of the retrospective tracking of information that will result in underreporting and misreporting of events. However, researcher short use precise, unambiguous, and well‐structured questions to help respondents understand exactly what they are being asked to recall to mitigating recall bias. It is important to note that the AORs reported in this study have wide confidence intervals. This reflects uncertainty in the estimates, due to low event counts in some categories. Therefore, while these associations are suggestive, they should be interpreted cautiously. Some variables like radiological finding, antibiotics, hospital admission and seasonal effect data were not included, so we recommend future researcher to include those variables.

## 6 Conclusion

The prevalence of CAP was different from district to district in Awi zone. Educational status of mother/caregiver/, stunting, age of child, history of having diarrhea in the last 2 weeks, family history of acute lower respiratory tract infection in the last 2 weeks, family history of acute upper respiratory tract infection in the last 2 weeks, fuel mainly used for cooking, child stays during cooking, and presence of separate kitchen for household were significantly associated with community acquired pneumonia among 2–59 months old children in Awi zone, northwest Ethiopia. Therefore, we recommend appropriate and adequate health education regarding nutritional intervention, prevention and early treatment of ARTI, and preventing indoor air pollution to reduce the risk of community acquired pneumonia.

## Author Contributions

N.A.B. contributed to design, supervised the data collection, performed the data analysis, interpreted the result, and drafted the manuscript. A.Y.W. supervised the data collection and evaluated the scientific content of the study. C.A.M. participated in designing the study, data analysis, and data interpretation. G.M.A. and Z.M. edit the manuscript. All authors have read and approved the final version of the manuscript.

## Consent

The authors have nothing to report.

## Conflicts of Interest

The authors declare no conflicts of interest.

## 1 Transparency Statement

The lead author Nigussie Adam Birhan affirms that this manuscript is an honest, accurate, and transparent account of the study being reported; that no important aspects of the study have been omitted; and that any discrepancies from the study as planned (and, if relevant, registered) have been explained.

## Data Availability

The data used to support the study findings are available from the corresponding author upon request.
